# Therapeutic Progress

**Published:** 1902-09

**Authors:** Nelson W. Wilson

**Affiliations:** Buffalo, N. Y.


### Therapeutic Progress.

EDITED BY NELSON W. WILSON, M. D., Buffalo, N.Y.

### TREATMENT OF ERYSIPELAS.

N. G., in the *Philadelphia Med. Jour.*, suggests this procedure in the treatment of erysipelas, which in spite of the author's initials is very good:

The affected area is first inclosed in a painted ring of tincture of iodine. The ring is not to be started at the margin of the reddened area, but from two to three inches from it, and a sufficient number of coats should be applied to cause a slight desquamation of the upper layers of the skin. At the same time the whole surface inclosed in the ring is to be covered with an ointment of ichthyol, about one dram to one or two ounces of vaseline. This is covered with a piece of gauze, and a hot stupe applied, and changed about every four hours. At the end of twelve hours the ichthyol ointment is washed off and a fresh coat applied, and if the iodine has not had sufficient effect, one or two more coats are applied.

---

### CARBOLIC ACID POISONING.

THE *Journal of Medicine and Science* says: "Whatever else you do in internal carbolic acid poisoning, give at once a large dose of alcohol—whiskey, brandy, rum or gin will answer—and repeat it often."

---

EXPERIMENTS WITH ADRENALIN.

ELSBERG, in *American Medicine*, gives a very comprehensive report of a series of experiments with adrenalin chlorid as an addition to solutions for local anesthesia. He says: "Adrenalin chlorid, which is the active blood pressure-raising principle of the suprarenal gland recently discovered and investigated by Dr. Takamine, is now on the market as an amorphous crystaline powder or in the form of a 1-1000 solution. It is a powerful astringent, so that a drop of a 1-10,000 solution will blanch the conjunctiva in from 30 to 60 seconds.

"Elsberg has been carrying on a series of experiments with this new drug and finds that if a drop of a 1-1000 solution be injected under the normal skin a slight burning sensation is felt but no anesthesia occurs. Within one minute an area of skin about two inches in diameter becomes blanched and almost bloodless and remains so from six to twelve hours. The same effect will be observed if a 1-5000 to 1-15,000 solution be used, but with these weaker solutions the blanching appears only after a few minutes and disappears after three to six hours. After the blanching of the skin disappears the tissue apparently returns to its normal condition. No deleterious effects such as sloughing or subcutaneous ecchymosis ever followed these injections. In the course of the investigations cocain and eucain solutions containing adrenalin in the proportion of 1-5000 to 1-20,000 were used. It was found that the anesthetic properties of the cocain and eucain were preserved while the adrenalin caused the same blanching of the tissues as previously observed, which extended one to two inches beyond the area infiltrated.

"In performing minor operations under cocain to which 1-5000 to 1-20,000 adrenalin had been added only the larger vessels bled when cut across. The smaller vessels were contracted so tightly that no blood could escape from them and therefore there was no oozing. It was unnecessary to sponge off the wound a single time during an operation. The healing of the wound was not interfered with in any way. Upon theoretical grounds it was expected that secondary hemorrhage would take place in from three to twelve hours as the effect of the drug passed off. This, however, has not been the case in the 30 cases operated upon. Experience with the drug is still small, and what will be the result in operations upon larger wounds remains to be determined.

"For small operations the addition of adrenalin chlorid is of distinct advantage in that it raises the blood pressure and overcomes the depressing effect of the cocain, at the same time it entirely does away with the oozing of blood from the wound."

In genitourinary work the writer has used adrenalin. It checks hemorrhage, but in several cases it was followed by secondary hemorrhage, rather free. Its use is now limited to circumcision in very young infants, and it is there applied in very weak solution when the open method is used.

### ICHTHYOL IN PULMONARY TUBERCULOSIS.

ICHTHYOL (*Merck's Archives*) has been employed frequently in the treatment of pulmonary tuberculosis, with gratifying results. Dr. A. Mostkoff (*Allg. Med. Central.-Zeit.*, 1901, No. 76) has used it for a period of more than two years in this disease, administering it diluted with an equal quantity of water, in doses of 5 to 20 drops, thrice daily, in wine or black coffee as a vehicle, after meals. Symptoms of intolerance on the part of the digestive organs were not observed, and the author concludes that ichthyol is perfectly non-toxic. Fifty cases all told, are recorded. The treatment was a distinct success. The appetite improved under the use of the remedy, the patients often gained in weight, the annoying night sweats were relieved, the cough was quieted and fever reduced.

Ichthyol, according to the author, may be recommended as an efficient substitute for creosote and its derivatives in the treatment of pulmonary tuberculosis.

### ACETANILID POISONING.

STEWART, F. T., (*Philadelphia Med. Jour.*) reports two cases emphasising the necessity of caution even when using acetanilid externally. The first patient had sustained an extensive burn of the left lower extremity. The doctor covered the raw surface with Thiersch's skin grafts taken from the right leg and thigh, and asked an assistant to dress the right limb while he completed the left. Early the next morning the patient became cyanotic, collapsed, and finally unconscious. On investigating the cause for such an alarming episode he learned that the right leg had been copiously dusted with acetanilid.

The second patient was a baby, aged four months, suffering from intertrigo of the scrotum, buttocks and thighs. A neighboring physician advised a liberal application of the following powder: calomel, ½ dr.; bismuth subnitrate and acetanilid, of each, 2 dr. The following day intense cyanosis developed. With the first case fresh in mind, the cause of trouble was easily found.

Both cases reacted readily. Stewart says that in aseptic cases acetanilid has no place; in septic cases there are more efficient, less dangerous agents at command.

---

### TREATMENT OF CHRONIC GASTRIC CATARRH.

EWALD (*International Medical Magazine*) says chronic gastric catarrh has undoubtedly two results which require therapeutic treatment: (1) Diminution in the production of HCl and pepsin, and (2) weakening of the motor function of the gastric musculature. For both we have an efficient treatment. Regarding (1), HCl should be prescribed in such quantities that the deficit will be fully compensated. One should give as large and as frequently repeated doses as possible. Ewald gives as many drops of dilute HCl in a half glass of water every ten minutes for three doses after meals as the patient can stand, *i.e.*, take without having a too sour taste in the mouth. A better way, if one cares to go that far, is to introduce a 0.02 per cent. HCl solution directly into the stomach through the stomach tube. He has given in this manner as much as 300 c.c. twice daily after the two larger meals. To increase motility of the gastric musculature, tincture of nux vomica stands first. In an infusion of cundurango bark (20 to 30 grams infused in 300 c.c. of water for twelve hours and evaporated to 150 c.c. by gentle heat) he puts 5 grams tincture of nux vomica and 5 grams dilute HCl. Massage, when performed by skilled hands—but only then—is valuable. Intragastric electricity is used whenever electricity is possible. One electrode is introduced into the well-washed stomach, the other over the belly. For stimulating influence, the faradic; for sedative, the galvanic (anode in the stomach) current four or five milliamperes, three to five minutes. He uses the common form of faradic apparatus with high tension and quick interrupter. The needle spray, preferably Einhorn's, is useful. To remove fermenting masses, wash out the stomach. To limit fermentation, the following:

| | | | |
|---|---|---|---|
| ℞ | Resorcini resublim . . . . . . . . . . . . . . . . | 5.0 | ( 75 gr.) |
| | Bismuth salicylat . . . . . . . . . . . . . . | 10.0 | (150 gr.) |
| | Natrii bicarbonat . . . . . . . . . . . . . . . | 15.0 | (225 gr.) |
| | Sacchari albi . . . . . . . . . . . . . . . . . . . . | 15.0 | (225 gr.) |

M. Sig.—Teaspoonful (small) every two hours.

The bowels must be regulated. The diet: patients should never eat until fully satisfied. Beverages should always be taken lukewarm, never too hot or too cold. The strong alcoholic and carbonated drinks should be prohibited. Proper care of mouth, teeth, etc., should not be neglected. Hard boiled eggs, stringy and tendinous meats, warm flesh, meat marbled with fat—sausage and the like—are all prohibited.

---

### MUSCULAR RHEUMATISM.

G. LYON (*Le Progrèss Médicale*) gives this prescription for muscular rheumatism:

| | | | |
|---|---|---|---|
| ℞ | Baume de Fioraventi . . . . . . . . . | c. c. 20.0 | (ʒvi |
| | Spt. camphoræ. . . . . . . . . . . . . . . | 20.0 | (ʒv) |
| | Spt. terebinth . . . . . . . . . . . . . . . . | 3.9 | (mxlv) |
| | Chloroformi. . . . . . . . . . . . . . . . . . | 5.0 | (mlxxv) |
| | Menthol . . . . . . . . . . . . . . . . . . . | gm. 2.0 | (gr. xxx) |

Apply with light friction.

---

### CHRONIC MALARIA.

IN CHRONIC malaria, M. Stephanopoulos uses this ointment twice a day, rubbed in with friction:

| | | | |
|---|---|---|---|
| ℞ | Iodoformi . . . . . . . . . . . . . . . . . . . | gm. 3.0 | (gr. xlv) |
| | Ext. belladon . . . . . . . . . . . . . . . . . | 4.0 | (ʒi) |
| | Ext. belladon . . . . . . . . . . . . . . . . . . | 4.0 | (℥ij) |
| | Ol. menth. pip . . . . . . . . . . . . . . . . . | gtt. xx. | |

For the general condition he gives two of these pills before dinner at noon and two before supper:

| | | | |
|---|---|---|---|
| ℞ | Ac. arsenosi . . . . . . . . . . . . . . . . . . | 0.001 | (gr. $\frac{1}{60}$) |
| | Iodoformi . . . . . . . . . . . . . . . . . . . | 0.06 | (gr. i) |
| | Ferri et potass. tart . . . . . . . . . . . . . . | 0.06 | (gr. i) |
| | Ext. bellad . . . . . . . . . . . . . . . . . . . | 0.03 | (gr. iss) |
| | Ext. quinquinæ . . . . . . . . . . . . . . . . . | 0.06 | (gr. i) |
| | Quin. sulphat . . . . . . . . . . . . . . . . . | 0.02 | (gr. $\frac{1}{3}$) |
| | Ext. nucis. vom . . . . . . . . . . . . . . . . | 0.01 | (gr. $\frac{1}{6}$ |
| | Ext. valerianæ . . . . . . . . . . . . . . . . . | 0.06 | (gr. i) |

For one pill.

### A REAL RHEUMATISM CURE.

LAYNE (*Cincinnati Lancet Clinic*) has been experimenting with salol, colchicum, the iodides and salicylates and has given them all up in favor of the prescription which he considers a specific:

| | | | | |
|---|---|---|---|---|
| ℞ | Ext. xanthoxylini | | ℥i.–ii. | 30.00–60.00 |
| | Ext. asclepiadis cornuti. | | | |
| | Ext. solani dulcamara | āā | ℥ss. | 15.00 |
| | Ext. taraxaci densleonis | | ℥ii. | 60.00 |
| | Spr. frumenti, q. s. ad. | | ℥viii. | 240.00 |

Mix. Dose—Four teaspoonfuls after each meal.

---

### INFANTILE BRONCHITIS.

THE *St. Louis Courier of Medicine* gives the following for infantile bronchitis:

| | | |
|---|---|---|
| ℞ | Tinct. nucis vomica | gtt. xvj |
| | Tinct. digitalis | gtt. xvj |
| | Liq. peptonoides cum creos | ʒiv |
| | Aque gaultheria | q. s. ad ℥ij |

M. Sig.—ʒj every four hours (for six months' child).

For cyanosis and cardiac inadequacy of infantile bronchitis: Mustard paste, 1 part mustard to 5 parts flour, warm water q.s. to make a paste; over entire chest until well irritated.

Also, internally.

| | | |
|---|---|---|
| ℞ | Spir. glonoini | gtt. iij |
| | Spir. frumenti | ʒij |
| | Aqua menth. pip | ʒij |
| | Aqua | q. s. ad ℥j |

M. Sig.—One-half teaspoonful p. r. n.

For infantile bronchitis, with symptoms of suffocation: Place child under tent and "steam" with beechwood creosote 5 to 20 drops to a quart of water. Keep under for twenty minutes every hour or two until marked improvement.

---

### PHARYNGITIS AND ACUTE BRONCHITIS.

IN laryngitis and acute bronchitis, Liègeois prescribes the following:

| | | | |
|---|---|---|---|
| ℞ | Sodii benzoat | gm. 4.0 | (ʒi) |
| | Tinct. aconit | c. c. 1.20 | (mxx) |
| | Aq. laurocerasi | 4.0 | (ʒi) |
| | Syr. tolutani. | | |
| | Syr. codeinæ. | | |
| | Aquæ | aa. 60.0 | (℥ij) |

M. Sig. To be taken in the twenty-four hours. For pharyngitis he gives large doses of sodium benzoate.

